# More Than a Case of Cellulitis: Pasteurella multocida Bacteremia

**DOI:** 10.7759/cureus.36096

**Published:** 2023-03-13

**Authors:** Zahra E Barsi, Jesse Allen, Armando Meza

**Affiliations:** 1 Division of Infectious Diseases, Texas Tech University Health Sciences Center El Paso, Paul L. Foster School of Medicine, El Paso, USA; 2 Division of Infectious Diseases, Texas Tech University Health Sciences Center El Paso, El Paso, USA

**Keywords:** clinician education, immunocompromised, bacteremia, cellulitis, pasturella multocida

## Abstract

*Pasteurella multocida* (*P. multocida*) is an anaerobic Gram-negative coccobacilli belonging to the Pasteurella genus. It is found in many animals' oral cavities and gastrointestinal tracts, including those of cats and dogs. In this case report, we present an individual with cellulitis of the lower extremity who was later found to have *P. multocida* bacteremia. The patient had four pet dogs and one pet cat. He denied obtaining any scratches or bites from the pets. The patient initially presented to an urgent care center complaining of a one-day history of proximal left lower extremity edema, erythema, and pain. He was diagnosed with left leg cellulitis and discharged home on antibiotics. Three days after the patient was discharged home from the urgent care center, blood cultures returned positive for *P. multocida*. The patient was then admitted for inpatient treatment with intravenous antibiotics. Clinicians should always ask about domestic and wild animal exposure, even in the absence of bites or scratches. In the immunocompromised patient presenting with cellulitis, clinicians should consider the possibility of *P. multocida *bacteremia in those with pet exposure.

## Introduction

*Pasteurella multocida (P. multocida) *is an anaerobic Gram-negative coccobacilli belonging to the *Pasteurella* genus. It is found in many animals' oral cavities and gastrointestinal tracts, including those of cats and dogs. Animals are often asymptomatic carriers, although they can become infected with *P. multocida* at times. Infections with *Pasteurella* often occur following contact with animal mucosal secretions. Therefore, animal bites, or scratches, are the most common source of *Pasteurella* infection [1-4]. Although it is generally more common to be bitten by a dog, cat bites are more likely to become infected. *P. multocida *is the most common organism isolated in soft tissue infections following dog and cat bites or scratches [3]. Infections following cat bites are caused by *P. multocida* in 75% of cases and 50% of infected dog bites [1,3]. Although the most common exposure humans can get is from domestic dogs and cats, *P. multocida *is present in other animals such as birds, rabbits, and swine. These animals can also be a source of infection [1].

The most common type of infection caused by *P. multocida* is skin and soft tissue infections [3]. More serious infections, including osteomyelitis, joint and intra-abdominal infections, bacteremia, endocarditis, and meningitis, are often seen in those with underlying comorbidities such as diabetes mellitus, cirrhosis, chronic obstructive pulmonary disease, hypertension, and malignancy among others [1,3,5]. Specifically, respiratory tract infections are more likely to be seen in those with underlying lung pathologies such as chronic obstructive lung disease and bronchiectasis [1,3]. The treatment of choice for local *P. multocida* infections is amoxicillin and clavulanic acid [3]. Other treatment options include cephalosporins, fluoroquinolones, metronidazole, and tetracyclines [1,3].

In this case report, we present an individual with cellulitis of the lower extremity who was later found to have *P. multocida* bacteremia. The patient had four pet dogs and one pet cat. He denied obtaining any scratches or bites from the pets. As reported by others in similar cases with no inciting bites or scratches from pets, we believe that respiratory droplets or saliva from the patient’s pets entered through the patient’s left lower extremity into the patient's circulatory system, leading to bacteremia [6,7].

## Case presentation

A 50-year-old male with a significant history of type 2 diabetes complicated by a history of multiple foot ulcers, hypertension, and cirrhosis from alcohol use presented to an urgent care center (UCC) complaining of a one-day history of proximal left lower extremity edema, erythema, and pain. He was also experiencing malaise, chills, and shortness of breath, which started earlier in the day. One week before his presentation, he reported a blister on the plantar area of the foot over just below the left first toe that had since ruptured and appeared to be healing well with no erythema or pain. A review of systems was negative for any other complaints.

**Figure 1:**
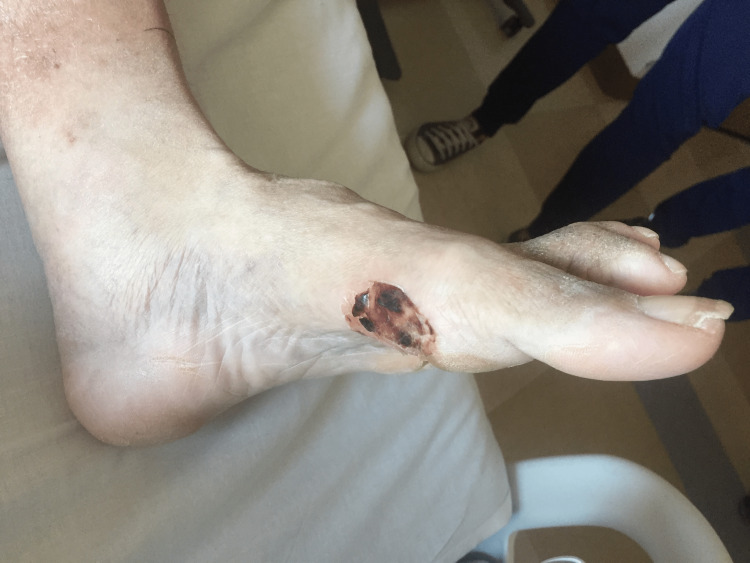
Left foot blister four days after presenting to the urgent care center

The physical exam was notable for a debriefed blister over the first metatarsophalangeal (MTP) joint without any signs of infection such as warmth, drainage, fluctuance, and erythema. There was notable erythema with warmth to touch and tenderness above the left medial ankle. Figure 1 displays the blister four days after presenting to the UCC.

Initial vital signs were notable for a temperature of 39.4° C, a pulse of 117 beats per minute, a respiratory rate of 16 breaths per minute, a blood pressure of 154/84 mmHg, and oxygen saturation of 95% on room air. Initial laboratory results were notable for a white blood cell count of 7.20 K/μL and a lactic acid of 1.6 mmol/L. The initial workup included X-ray imaging of the left lower leg and foot, which showed no acute osseous process.

The patient received intravenous (IV) piperacillin/tazobactam (Zosyn) 4.5 g and vancomycin 2,000 mg after blood cultures and wound cultures of the left foot were collected. Subsequently, in the UCC, his vitals were stable. He was diagnosed with left leg cellulitis and discharged home on oral trimethoprim/sulfamethoxazole for 10 days with instructions to follow up with his primary care provider in two days.

Three days after the patient was discharged home, blood cultures returned positive for P*. multocida.* The wound culture of the left toe grew only skin and environmental flora. The patient was contacted and referred to the hospital for inpatient admission and treatment with IV antibiotics.

On admission, initial vitals were notable for a temperature of 37.4° C, a pulse of 85 beats per minute, a respiratory rate of 18 breaths per minute, a blood pressure of 154/84 mmHg, and oxygen saturation of 95% on room air. The laboratory findings were notable for a white blood cell count of 3.80 K/μL, a C-reactive protein of 4.29 mg/dL, an erythrocyte sedimentation rate of 98 mm/hr, and creatinine of 1.2 mg/dL. A repeat X-ray of the left foot showed no evidence of osteomyelitis. Hemoglobin A1C was found to be 7.8.

The patient was started on ceftriaxone one gram intravenously. Further diagnostic workup included a two-dimensional echocardiogram that showed no evidence of endocarditis and a lower extremity venous Doppler, which was negative for a deep vein thrombosis. After the initiation of antibiotic therapy, blood cultures were negative for growth. The patient was discharged six days after admission on IV ceftriaxone 2 gm once daily for a total of two weeks, followed by oral amoxicillin-clavulanic acid 875 mg to 125 mg every 12 hours for two more weeks.

## Discussion

*Pasteurella* bacteremia should be considered in individuals with soft tissue infections, especially those with pet exposure and underlying comorbidities such as diabetes, cirrhosis, hypertension, malignancy, and immunosuppressed states [5,8,9]. It is important to note that individuals do not need to have a reported history of bites or scratches from animals, as bacteremia has been seen in those with no reported history of traumatic pet contact [6]. Clinicians must diligently inquire about all animal contact a patient has had, and not necessarily just animal-inflicted injuries [2].

A retrospective study found that patients with non-bite infections of *P. multocida* are more likely to present with bacteremia, have increased morbidity, become hospitalized, have comorbidities, and be immunocompromised [10]. In immunocompromised individuals, healthcare providers should make patients aware of the potential risk of this infection from pet exposure [10]. Immunocompromised individuals should limit contact with pets that could potentially lead to bites or scratches. In a review of cases of *Pasteurella* bacteremia, 68.1% of patients had another documented source of infection; of these patients, 20.2% had cellulitis [8].

Although *P. multocida* bacteremia is rare, there is a high rate of mortality associated with bacteremia. Mortality from *Pasteurella* bacteremia has been reported to be between 14% and 31% [8]. Therefore, it is important to be vigilant about suspecting potential bacteremia in those who are immunocompromised early in their clinical course.

## Conclusions

The key takeaways from this case are that clinicians should always ask about domestic and wild animal exposure, even in the absence of bites or scratches. In the immunocompromised patient presenting with cellulitis, consider the possibility of *P. multocida* bacteremia in those with pet exposure. In an outpatient setting, consider ordering blood cultures to rule out *P. multocida* bacteremia, especially in those with cellulitis, underlying comorbidities, recent animal exposure, and fever.
